# 

**Published:** 1917-01

**Authors:** 


### CONTENTS.

ORIGINAL COMMUNICATIONS.................411

EDITORIAL.................423

SELECTIONS AND ABSTRACTS.................425

BOOK REVIEW.................437

